# Agreement between Future Parents on Infant Feeding Intentions and Its Association with Breastfeeding Duration: Results from the *Growing Up in New Zealand* Cohort Study

**DOI:** 10.3390/ijerph15061230

**Published:** 2018-06-11

**Authors:** Emma J. Marks, Cameron C. Grant, Teresa Gontijo de Castro, Dinusha K. Bandara, Clare Wall, Susan M. B. Morton

**Affiliations:** 1 Growing Up in New Zealand, University of Auckland, Auckland 1142, New Zealand; cc.grant@auckland.ac.nz (C.C.G.); teresagcastro108@gmail.com (T.G.d.C.); Dinusha.Bandara@aifs.gov.au (D.K.B.); s.morton@auckland.ac.nz (S.M.B.M.); 2 Centre for Longitudinal Research-He Ara ki Mua, University of Auckland, Auckland 1142, New Zealand; c.wall@auckland.ac.nz; 3 Department of Paediatrics: Child and Youth Health, University of Auckland, Auckland 1142, New Zealand; 4 General Paediatrics, Starship Children’s Hospital, Auckland 1142, New Zealand; 5 Australian Institute of Family Studies, Melbourne, Victoria 3006, Australia; 6 School of Medical Sciences, University of Auckland, Auckland 1142, New Zealand

**Keywords:** breastfeeding, pregnant, partners, intentions, duration

## Abstract

Maternal intentions are believed to have the strongest influence on infant feeding. However, what has rarely been studied, are the associations of maternal and partner intentions, and the influence these factors have on infant feeding. Our objective was to describe breastfeeding intentions of pregnant women and their partners, agreement about these intentions, and whether this agreement is associated with breastfeeding initiation and duration. This study was completed within the *Growing Up in New Zealand* study. Agreement between mothers and partners on intended initial infant feeding method was fair (κ = 0.21, 95% confidence interval (CI) 0.17–0.25) as was intended breastfeeding duration (κ = 0.25, 95% CI 0.22–0.28). Infants whose parents agreed antenatally on breastfeeding only were more likely to have been breastfed for >6 months, after adjustment for maternal (odds ratio (OR) = 6.3, 95% CI 3.9–10.2) and partner demographics (OR = 5.7, 95% CI 3.6–9.2). Likewise, infants whose parents agreed antenatally to breastfeed for >6 months were more likely to have been breastfed for >6 months, after adjustment for maternal (OR = 4.9, 95% CI 3.9–6.2) and partner demographics (OR = 5.0, 95% CI 4.0–6.3). Interventions that promote breastfeeding to both mothers and partners which enable parents to reach agreement about intended feeding methods have the potential to increase both breastfeeding initiation and duration.

## 1. Introduction

Breastfeeding is the optimal method for providing nutrition that enables healthy infant growth and development [1]. In their review of the optimal duration of breastfeeding, the World Health Organization [1] found that the nutritional requirements of four to six month old infants are met by exclusive breastfeeding and that there was no evidence for nutritional disadvantage to babies being breastfed up to six months in either developed or developing countries.

Despite this, worldwide, no more than 35% of infants are exclusively breastfed during their first four months of life [1]. In developed countries, initiation rates of breastfeeding are high but there is then a rapid drop off in breastfeeding rates prior to six months of age. For example, in the United States (US) in 2012–13, breastfeeding was initiated in 79% but only 49% were still being breastfed at age six months [2]. Similar breastfeeding patterns have been described from Australia: 96% initiation, 69% breastfed at age four months [3]; the United Kingdom: 81% initiation, 34% breastfed at age six months [4]; and *Growing Up in New Zealand* (NZ) data shows 97% initiation, with 66% breastfed to six months [5].

Parental, and particularly maternal, intentions are believed to be the strongest influences on breastfeeding initiation and duration [6,7,8] and likely confound observed relationships of other maternal demographic, health and behavioural factors with breastfeeding initiation and duration [7]. A woman’s breastfeeding intentions are influenced by the support received from and the attitudes of the mother’s partner to breastfeeding [9,10,11,12,13,14] and also by the woman’s perception of her partner’s attitude towards breastfeeding [15,16]. Little is known about how a partners feeding intentions effect infant feeding practices or how partner’s perceive their influence with regards to feeding intentions and practices [17].

The true relationship of agreement between the mother and her partner on their breastfeeding intentions also requires the mother and her partner to be interviewed independently of one another. To date, such prospective investigation has been limited to a small (*n =* 112), convenience sample of low-income pregnant w omen and their partners in Honolulu [18]. This study used the Iowa Infant Attitude Scale (IIFAS) and showed higher IIFAS scores were significantly associated with mothers’ intention to breastfeed and that the parental scores were both highly correlated and equally important in their association with intent to breastfeed.

Utilising a cohort study into which enrolment of both mothers and partners occurred during pregnancy and each mother–partner pair were interviewed separately, our objectives were to (1) describe the intended infant feeding method and breastfeeding duration of pregnant women and their partners; (2) assess agreement between mothers and partners; (3) examine the socio-demographic characteristics associated with intended feeding method and duration and (4) determine whether agreement between pregnant women and their partners on initial infant feeding was associated with breastfeeding duration.

## 2. Methods

### 2.1. Study Design

We completed our study within New Zealand’s contemporary cohort study *Growing Up in New Zealand* (www.growingup.co.nz). *Growing Up in New Zealand* enrolled a cohort of pregnant women, with an estimated delivery date (EDD) between 25 April 2009 and 25 March 2010, and their partners who were residing in a geographically defined area of NZ chosen for its ethnic and socio-economic diversity. There were no other inclusion or exclusion criteria [19]. Eleven percent of all births in NZ during the study interval were enrolled. The *Growing Up in New Zealand* cohort is broadly generalisable to the national birth cohort [19,20]. Ethics approval was obtained from the Ministry of Health Northern Y Regional Ethics Committee (NTY/08/06/055), and written informed consent from all enrolled women and their partners.

### 2.2. Study Population and Sample

Six-thousand-eight-hundred-and-twenty-two pregnant women consented to their children participating in *Growing Up in New Zealand* [21]. With the consent of each enrolled woman, we approached partners independently, and obtained consent for the participation of 4401 partners. Over 99% reported they were the biological father of the cohort child/children [22].

### 2.3. Data Collection

Data were collected using face-to-face computer assisted personal interviews (CAPI). Data reported in this study include that obtained from CAPIs completed during pregnancy and infancy. The first CAPI was completed with the pregnant woman (most often in the last trimester of her pregnancy) and with her partner independently, typically in their own home [19]. Likewise, the second CAPI took place when the cohort children were nine months old [19]. This nine month interview was completed by 94% of mothers (*n =* 6384) and 93% of partners (*n =* 4094) [19].

### 2.4. Measurements

#### 2.4.1. Infant Feeding Intentions

To determine the true relationship of mothers’ and partners’ breastfeeding intentions with breastfeeding practices requires these initial intentions to be determined before the child is born. Questions and answer options for both mother and partner can be seen in Box 1. For intended breastfeeding duration, both mothers and partners were asked how long they thought was best (mothers) or appropriate (partners) to breastfeed their baby. The question was designed to gauge ideal breastfeeding duration from both parents (matched data). How long mothers would like to breastfeed for was asked in a subsequent question. The difference between the number of mothers whose ideal breastfeeding duration was longer than six months and those who would like to breastfeed for longer than six months was however less than 1%. In order to be able to compute matched pair data for breastfeeding duration the ideal breastfeeding questions (Box 1) were used.

Box 1 **Questions asked of pregnant women and their partners about infant feeding intentions and of the women when their children were infants about breastfeeding that occurred during infancy.**

**Questions Asked during Pregnancy of Both Pregnant Women and Their Partners about Infant Feeding Intentions**

**Questions Asked during Infancy of Mothers Only about Breastfeeding Initiation and Duration**
1. How are you intending to feed your baby when they are first born? (Pregnant women) Or How would you prefer this baby to be fed when they are first born? (Partners)BreastBottleBoth breast and bottleHaven’t decided
2. How long do you think is best to breastfeed your baby? (Pregnant women) Or How long do you think is appropriate to breastfeed a baby? (Partners)Up to 6 weeksUp to 3 monthsUp to 6 monthsLonger than 6 monthsHaven’t thought about this (Partners only)
1. Did you ever breastfeed this baby? Breastfeeding includes feeding expressed milk.Yes, I am breastfeeding (includes supplementing with formula and solids)Yes, I breastfed but have stopped nowNo, I have never breastfed
2. How old was your baby when you stopped breastfeeding? This refers to any breastfeeding, whether exclusive or not.MonthsWeeksDays

#### 2.4.2. Infant Feeding Outcomes

World Health Organization (WHO) definitions of any breastfeeding initiation and duration were used [23] to formulate the questions asked at the nine month mother interviews (Box 1). When calculating breastfeeding duration those infants never breastfed were included in the analysis.

#### 2.4.3. Maternal and Partner Demographics

Demographic data collected from both pregnant women and their partners antenatally included: self-identified ethnicity; age; highest educational level attained; annual household income; household socioeconomic deprivation [24]; and self-reported health status. Individuals who identified more than one ethnicity were asked to self-prioritise these multiple ethnicities. Household socioeconomic deprivation was defined using an area level measure, the New Zealand (NZ) Index of Deprivation [24]. NZDep06 combines nine socioeconomic characteristics from 2006 census data collected at aggregations of approximately 100 people and assigned to individual households based on geo-coded address data [24]. General health status was asked of both mother and partner in order to determine if there was an association between self-rated health and infant feeding. It was scored on a five-point scale from excellent to poor [25]. Pregnant women were asked to describe their parity (first or subsequent child) and whether the pregnancy was planned. 

### 2.5. Data Analysis 

Agreement between pregnant women and partners on intended feeding methods and breastfeeding duration were described using weighted Kappa (κ) with 95% confidence intervals (CI). In order to match categories for breastfeeding duration the option “Haven’t thought about this” was excluded from the analysis as it was only asked of partners (see Box 1). Agreement was categorised as ‘poor’ (κ ≤ 0.2), ‘fair’ (>0.2 to 0.4), ‘moderate’ (>0.4 to 0.6), ‘good’ (>0.6 to 0.8) or ‘very good’ (>0.8 to 1.0) [26].

The relationship between the demographic characteristics of the pregnant women and of their partners with their intentions for infant feeding method and their intentions for breastfeeding duration were investigated using logistic regression. For the purposes of analyses, we dichotomised intended and actual infant feeding (solely breastfed versus all other feeding method options) and intended and actual breastfeeding duration (≤6 months versus >6 months). 

The independence of associations between the initial intended feeding method and intended breastfeeding duration of mothers and partners with the actual feeding method and actual breastfeeding duration were determined using multivariable logistic regression. These models included, as potential confounders, self-prioritised ethnicity; age; educational level; child parity (mother only); pregnancy status (mother only); self-reported health status; household income; and household socioeconomic deprivation. Multivariable logistic regression models were then used that included variables describing the same confounding variables to determine the associations between the concordance and discordance of mothers and partners on their intended feeding method and breastfeeding duration with actual feeding method and breastfeeding duration. Associations were reported using adjusted odds ratios (OR) and 95% confidence intervals (CI). All analyses were conducted using SAS software (version 9.3, SAS Institute, Cary, NC, USA). A *p* Value of <0.05 was considered statistically significant.

## 3. Results

Responses on intended feeding method were provided by 6181 (91%) and 4149 (94%) and on intended breastfeeding duration by 5986 (88%) and 4069 (93%) of the 6822 enrolled mothers and 4401 enrolled partners, respectively. Paired (matched) demographic information from both mothers and partners were available for 4397 of the 6822 women (65%) and 4397 of the 4401 partners that were enrolled. Responses on intended feeding method were provided by 4155 (95%) and 4146 (94%) and on intended breastfeeding duration by 4060 (92%) and 4066 (94%) of the 4397 matched pairs of mothers and partners respectively (Table 1).

Within the matched pairs of mothers and partners, a significantly larger proportion of partners than mothers remained undecided antenatally about intended feeding method (2% vs. 1%, *p* < 0.001). Among those that had decided, the proportion decided upon only breastfeeding was greater for mothers than partners (91% vs. 89%, *p* < 0.001). A larger proportion of mothers than partners intended that breastfeeding duration would be greater than six months (68% vs. 51%, *p* < 0.001) (Table 1).

Eighty-two percent of the mother–partner pairs were in agreement on breastfeeding as the initial intended feeding method for their expected infant. Agreement between mother and partner on intended initial infant feeding method was fair (κ = 0.21, 95% CI 0.17–0.25). Forty-two percent of the mother–partner pairs were in agreement that that their expected infant should be breastfed for >6 months. Agreement between mother and partner for intended breastfeeding duration was also fair (κ = 0.25, 95% CI 0.22–0.28) (Table 2).

### 3.1. Relationships of Maternal Demographic Characteristics with Intended Infant Feeding Method and Breastfeeding Duration 

In univariate analyses, maternal ethnicity, parity, pregnancy planning, and health status were associated with both breastfeeding being their intended initial infant feeding method and their intended breastfeeding duration being >6 months (Table S2). Maternal education and household socioeconomic deprivation were associated with breastfeeding being their intended initial infant feeding method and maternal age with their intended breastfeeding duration being >6 months (Table S2).

Pregnant women were less likely to intend that breastfeeding only would be their initial infant feeding method if they were of Māori (OR = 0.66, 95% CI 0.47–0.94), Pacific (OR = 0.42, 95% CI 0.31–0.57), or Asian (OR = 0.56, 95% CI 0.42–0.74) compared with European ethnicity; or if they had no secondary (OR = 0.43, 95% CI 0.29–0.65) or only secondary education (OR = 0.68, 95% CI 0.53–0.86). Pregnant women were more likely to intend that breastfeeding only would be their initial infant feeding method if this was their first child (OR = 1.26, 95% CI 1.01–1.56); if this pregnancy was planned (OR = 1.38, 95% CI 1.10–1.71); or if they were in very good (OR = 1.57, 95% CI 1.22–2.02) or excellent self-reported health (OR = 1.41, 95% CI 1.05–1.89) (Table 3).

Pregnant women were less likely to intend to breastfeed for >6 months if aged < 20 years (OR = 0.35, 95% CI 0.24–0.50) or 20 to 29 years (OR = 0.82, 95% CI 0.71–0.94) versus 30 to 39 years; or if they had no secondary education (OR = 0.66, 95% CI 0.48–0.91); or if this was their first child (OR = 0.53, 95% CI 0.46–0.60). Pregnant women were more likely to intend to breastfeed for >6 months if this pregnancy was planned (OR = 1.19, 95% CI 1.03–1.37) or if they were in excellent self-reported health (OR = 1.29, 95% CI 1.07–1.55) (Table 3).

### 3.2. Relationships of Partners’ Characteristics with Intended Initial Infant Feeding Method and Breastfeeding Duration

In univariate analyses, the partner’s education was associated with breastfeeding being their intended initial infant feeding method (Table S2). Partner’s ethnicity, age, household income, and household deprivation were all associated with their intended breastfeeding duration being >6 months (Table S2). 

Partners were less likely to intend that breastfeeding only would be their initial infant feeding method if of Pacific (OR = 0.71, 95% CI 0.55–0.93) versus European ethnicity; or <20 (OR = 0.51, 95% CI 0.30–0.89) versus 30 to 39 years old; or had no secondary education (OR = 0.82, 95% CI 0.71–0.94). In comparison with partners living in the most deprived quintile of households (deciles 9–10), those living in households in deciles 5–6 (OR = 1.34, 95% CI 1.01–1.78) or 7–8 (OR = 1.36, 95% CI 1.03–1.80) were more likely to intend to breastfeed (Table 4).

Partners were less likely to intend that their infant would be breastfed for >6 months if <20 (OR = 0.28, 95% CI 0.16–0.48) versus 30 to 39 years old; or they were living in a household in the least (OR = 0.70, 95% CI 0.57–0.86) or second least deprived quintile of all NZ households (OR = 0.73, 95% CI 0.60–0.89). Partners were more likely to intend that their infant would be breastfeed for >6 months if of Māori (OR = 1.48, 95% CI 1.17–1.86), Pacific (OR = 1.51, 95% CI 1.22–1.88), Asian (OR = 1.69, 95% CI 1.39–2.06), or Other (OR = 1.61, 95% CI 1.18–2.21) compared with European ethnic groups; or in excellent compared with good self-reported health (OR = 1.32, 95% CI 1.07–1.64) (Table 4).

### 3.3. Comparison of Antenatal Feeding Intentions with Actual Feeding Outcomes when Infant was Aged Nine Months (Table 5)

At the interview completed when their infant was nine months old, 3016 (47%) of mothers stated they were still breastfeeding their child, 3222 (50%) mothers had breastfed their child but now stopped and 207 (3%) of mothers had never breastfed their child. At this interview, 5122 (99%) of the women who intended to breastfeed were still breastfeeding or had breastfed but now stopped. In comparison, 464 (93%) of the women who intended to both breast and bottle feed, 41 (38%) of the women who intended to bottle feed their child, and 25 (76%) of the women who were undecided antenatally were either still breastfeeding or had breastfed but now stopped.

When compared with infants of parents who were in agreement on other infant feeding method intentions, those infants whose parents agreed antenatally on only breastfeeding were more likely to have been breastfed for >6 months, with these associations evident after adjustment for maternal (OR = 6.3, 95% CI 3.9–10.2) or partner demographics (OR = 5.7, 95% CI 3.6–9.2). In comparison with infants of parents who were in agreement antenatally on a shorter duration of breastfeeding, infants whose parents agreed antenatally to breastfeed for >6 months were more likely to have been breastfed for >6 months, after adjustment for maternal (OR = 4.9, 95% CI 3.9–6.2) or partner demographics (OR = 5.0, 95% CI 4.0–6.3).

Infants whose parents agreed antenatally on only breastfeeding were more likely to have been breastfed for >6 months compared to those parents with all other feeding intentions (either concordant or discordant), after adjustment for maternal (OR = 2.8, 95% CI 2.3–3.3) or partner demographics (OR = 2.6, 95% CI 2.1–3.0). Additionally, an infant was more likely to have been breastfed for >6 months if both parents were in concordance antenatally to breastfeed their infant for >6 months compared to those infants whose father intended to feed for ≤6 months, adjusted for maternal (OR = 3.5, 95% CI 2.9–4.1) or partner demographics (OR = 3.2, 95% CI 2.8–3.8); or whose mother intended to feed for ≤ six months, adjusted for maternal (OR = 5.0, 95% CI 4.2–5.9) or partner demographics (OR = 4.7, 95% CI 3.9–5.5).

## 4. Discussion

### 4.1. Summary of Principal Findings

In this study, sole breastfeeding was the intended method of feeding for the majority (89% of women and 87% of partners) of prospective parents. In contrast, larger differences were evident between women and their partners in their antenatal breastfeeding duration intentions, with 68% of women and 51% of their partners intending that the breastfeeding duration be >6 months. There was only a fair degree of agreement between individual women–partner pairs on their intentions for both method of feeding (κ = 0.21) and intended duration of breastfeeding (κ = 0.25). 

After adjustment for maternal and partner demographics, the infants of parents who were in agreement on breastfeeding as the initial infant feeding method were more than twice as likely to be breastfed, and the infants of parents who were in agreement on breastfeeding duration of >6 months were at least three times more likely to have been breastfed past 6 months of age.

### 4.2. Strengths and Weaknesses of the Study

Our study included a large and heterogeneous sample of prospective parents. In contrast, other studies that have investigated the breastfeeding intentions of both mothers and partners, for example Mitchell-Box et al. [18], have enrolled smaller samples (*n* ≈ 100) of mothers and partners derived from a specific sector of the socioeconomic spectrum. We were able to consider the intentions of both future mothers and their partners and were careful to obtain these independently of one another [19]. The reduction by 35% of mothers in the cohort in the matched paired data may have reduced the variability in some socio-demographic variables however the mothers and partners were still an ethnically and socioeconomically diverse sample (Table S1). Additionally, our retention rate at age nine months was high for mothers (94%) and partners (93%), thus reducing the bias introduced by variance in attrition between population subgroups. 

We did not use exclusive breastfeeding as a benchmark for measuring breastfeeding duration as we were more interested in overall initiation and duration. Whilst acknowledging that this prevents us from making direct comparisons with studies who have used this definition, our definition of breastfeeding is more consistent with the contemporary developed world reality where, although the global average percentage of exclusive breastfeeding (EBF) up to six months sits at 38% [28], EBF in developed countries is significantly lower and has not increased substantially despite several global initiatives (i.e., the Baby Friendly Hospital Initiative) [29]. For example, using data from the WHO database, the prevalence of exclusive breastfeeding up to 6 months is 18.6% in Australia (1995–6, *n =* 3252), 13.6% in the United States (2007–8, *n =* 16,985), 9% in Norway (2006, *n =* 3000), and 12.3% in Sweden (2007, *n =* 106,087) [30]. New Zealand’s population data is incomplete but the estimate from our cohort is that 16% were EBF to age six months [5]. 

The possibility of biased recall of breastfeeding duration is present in our study as the interview at which breastfeeding duration was described occurred when the infants were nine months old. However, previous studies have shown that maternal recall of breastfeeding initiation and duration offers a valid and reliable estimate for recall periods of three years or less [31].

### 4.3. Strengths and Weaknesses in Relation to Other Studies, Discussing Particularly Any Differences in Results

Highlighted by our study was the variance in influence of parental ethnicity, with maternal ethnicity associated with intended feeding method but not breastfeeding duration, whereas paternal ethnicity was associated with intended breastfeeding duration but not with feeding method. Other studies have investigated the association of ethnicity with breastfeeding initiation and duration (e.g., [6,32]). However, data investigating this relationship for both mothers and fathers is largely absent from the literature. Younger parental age and lower levels of educational attainment had an effect on intended feeding method and breastfeeding duration. This is comparable with published data reported from studies performed in both Australia and the United States [6,32]. Mother’s parity was shown to be both a positive and negative influence on breastfeeding. Women expecting their first child were more likely to intend to breastfed but less likely to intend to breastfed for more than six months. The effect of parity on breastfeeding has been shown in other studies, for example, Grummer-Strawn et al. [32]. In contrast to parity, pregnancy planning had a more consistent relationship with breastfeeding with both intention to breastfeed and breastfeeding duration positively associated with this being a planned pregnancy (see [5,33,34]). 

We also showed the association of parental health status with feeding intentions and breastfeeding duration. Mothers reporting they had very good or excellent health and partners reporting they had excellent health were more likely to intend to breastfeed and to breastfeed for longer than 6 months. Most research that has looked at predictors of breastfeeding and maternal health have focussed on specific health indicators such as maternal obesity (i.e., [35] or pregnancy health related conditions such as diabetes (i.e., [36]). We have shown self-reported maternal and paternal health status also to be a useful indicator of intended breastfeeding behaviours. More comprehensive research into the effects of parental health on infant feeding intentions and outcomes is needed.

Income in relation to breastfeeding attitudes and outcomes has been studied with conflicting results with some research finding positive correlations with breastfeeding intentions [37] and some inverse relationships [32]. Our study found no effect of maternal income or SES on intentions to breastfeed however we did find partners living in the most deprived quintile of households was associated with increased odds of intending that their child be breastfed; whereas living in the least deprived quintile of households or having a household income in the highest bracket (>$150,000) was associated with decreased odds of intending that their child be breastfed for >6 months. While it has been shown that partners’ knowledge and attitudes have an impact on maternal breastfeeding decisions and subsequent duration [10], there is little published research on the effect of partner socioeconomic status and income levels on infant feeding outcomes. 

### 4.4. Meaning of the Study: Possible Mechanisms and Implications for Clinicians or Policymakers

Our data shows that the antenatal period is a key time point to intervene if breastfeeding duration rates are to be increased and that any such intervention should include both the pregnant women and their partners. In terms of their positive influence on breastfeeding outcomes, multi-component interventions that span both the antenatal and postnatal periods are more effective than those that, for example, only focused on the antenatal time point or used only a single communication strategy (see reviews by [8,38]).

Numerous studies conducted in both developed and developing nations have shown the importance of the partners’ support of and positive attitudes towards breastfeeding as a determinant of breastfeeding initiation and duration [9,10,11,12,13,14]. Interventions that have included partners, either antenatally or postnatally, have been effective at increasing breastfeeding initiation and extending breastfeeding duration [14,16,39,40,41].

Maternal intentions, however, remain the strongest independent influence on breastfeeding initiation and duration [6,7,8]. Consequently, to be effective, interventions and policy that seek to promote maternal intentions to breastfeed need to be applicable to those women whose demographics place them at higher risk of not initiating breastfeeding or of breastfeeding for a shorter duration. Several studies have developed and assessed interventions within typically vulnerable subgroups of the population i.e., younger mothers [42] and those with less education (i.e., [43]). Our study findings indicate a need to also develop and assess interventions targeted towards mothers in poor health and mothers with previous children.

### 4.5. Unanswered Questions and Future Research

Our study highlights that parental agreement on breastfeeding intentions is important to both the infant feeding method chosen and the duration of breastfeeding. What has not been addressed by our study was what the mother or partner’s perceptions of each other were with regard to their intended feeding method or breastfeeding duration. The perceptions, for example, by the mother of her partner’s intentions with respect to infant feeding method, can be very inaccurate. This may have a negative effect on a perceived maternal support for breastfeeding and ultimate breastfeeding duration [15,16]. The negative perception by men of breastfeeding, especially in public, has also be been shown to affect the breastfeeding decisions made by the mother. Interventions focussed on fathers/men’s attitudes have been suggested as a way to increase breastfeeding rates and duration [40,44,45].

It will be important to determine why agreement between mothers’ and partners’ feeding intentions was only fair and to identify potential strategies that seek to increase agreement. There is a need to understand what factors determine breastfeeding intentions within ethnic groups. That intention to breastfeed varied with maternal ethnicity and intended duration of breastfeeding with paternal ethnicity suggest that these are two distinct areas for development research. Likewise, why partners of higher socioeconomic status mothers are less likely to intend for their infant to be breastfed for >6 months needs to be understood. The potential adverse effect of higher paternal income on breastfeeding is a contemporary issue that requires focussed attention.

## 5. Conclusions

Exactly when antenatal infant feeding method decisions are made by either mother or partner has not been fully investigated, thus the optimal time period to target interventions to make them most efficacious requires additional study. In order to create better public health promotion messages and increase the rates of breastfeeding and increase breastfeeding duration in line World Health Organizations Global Strategy for Infant Feeding [1], we need to further understand the context of infant feeding method decisions and how we can help parents to reach consensus on their intentions to breastfeed and to continue this until the infant is at least six months old.

## Figures and Tables

**Table 1:** Pregnant women and their partner’s intentions for infant feeding.

**Infant Feeding**	**All Pregnant Women and Partners**			**Matched Pairs of Pregnant Women and Their Partners**		
	Women	Partners	*p* value	Women	Partners	*p* value
	*n*_1_ = 6822	*n*_2_ = 4401		*n*_1_ = 4397	*n*_2_ = 4397	
	*n* (Col %)	*n* (Col %)		*n* (Col %)	*n* (Col %)	
Intended infant feeding method	*n*_1_ = 6181	*n*_2_ = 4149		*n*_1_ = 4155	*n*_2_ = 4146	
Breast	5481 (89)	3616 (87)	<0.001	3775 (91)	3613 (87)	<0.001
Both breast and bottle	533 (9)	416 (10)		305 (7)	416 (10)	
Bottle	130 (2)	51 (1)		51 (1)	51 (1)	
Haven’t decided	37 (1)	66 (2)		24 (1)	66 (2)	
Intended infant breastfeeding duration	*n*_1_ = 5986	*n*_2_ = 4069		*n*_1_ = 4060	*n*_2_ = 4066	
Up to 6 weeks	40 (1)	53 (1)	<0.001	20 (0)	53 (1)	<0.001
Up to 3 months	223 (4)	245 (6)		129 (3)	245 (6)	
Up to 6 months	1647 (27)	1295 (32)		1136 (28)	1294 (32)	
Longer than 6 months	4076 (68)	2072 (51)		2775 (68)	2070 (51)	
Haven’t thought about this	-	404 (10)		-	404 (10)	

- Not asked of mothers.

**Table 2:** Agreement between pregnant women and their partners about their intentions for their infant’s feeding method ^a^ and their intentions for their infant’s breastfeeding duration ^b^.

Pregnant Women’s Intentions for Their Infant’s Feeding Method	Partners Intentions for Their Infant’s Feeding Method							
	Breast *n* (%)	Bottle *n* (%)	Both Breast and Bottle *n* (%)		Haven’t Decided *n* (%)			**Total *n* (%)**
Breast	3381 (82)	21 (0)	311 (7)		53 (1)			3766 (91)
Both breast and bottle	202 (5)	<10 (0)	88 (2)		<10 (0)			305 (7)
Bottle	20 (0)	22 (0)	<10 (0)		<10 (0)			51 (1)
Haven’t decided	10 (0)	<10 (0)	10 (0)		<10 (0)			24 (1)
Total	3613 (87)	51 (1)	416 (10)		66 (2)			4146 (100)
**Pregnant Women’s Intentions for Their Infant’s Breastfeeding Duration**	**Partners Intentions for Their Infant’s Breastfeeding Duration**							
	**Up to 6 Weeks *n* (%)**		**Up to 3 Months *n* (%)**	**Up to 6 Months *n* (%)**		**Longer than 6 Months *n* (%)**	**Haven’t Thought about This *n* (%)**	**Total *n* (%)**
Up to 6 weeks	<10 (0)		<10 (0)	<10 (0)		<10 (0)	<10 (0)	19 (0)
Up to 3 months	<10 (0)		24 (1)	54 (1)		32 (1)	14 (0)	127 (3)
Up to 6 months	22 (1)		101 (2)	518 (13)		337 (8)	133 (3)	1111 (28)
Longer than 6 months	22 (1)		112 (3)	697 (17)		1665 (42)	242 (6)	2738 (68)
Total	48 (1)		238 (6)	1276 (32)		2041 (51)	392 (10)	3995 (100)

Abbreviations: CI, confidence interval. ^a^ Agreement on feeding intentions 84% (95% CI 83–85%); inter-rater agreement (weighted Kappa) 0.21 (95% CI 0.17–0.25). ^b^ Agreement on breastfeeding duration 55% (95% CI 54–57%); inter-rater agreement (weighted Kappa) 0.25 (95% CI 0.22–0.28).

**Table 3:** Independent associations of the demographics of pregnant women with their intended infant feeding method and intended infant breastfeeding duration.

Maternal Demographic Characteristics	OR (95% CI)	*p* Value	OR (95% CI)	*p* Value
Self-prioritised ethnicity ^a^		<0.001		0.08
European	1.00		1.00	
Māori	0.66 (0.47–0.94)		1.18 (0.94–1.48)	
Pacific Peoples	0.42 (0.31–0.57)		1.28 (1.00–1.63)	
Asian	0.56 (0.42–0.74)		0.92 (0.77–1.11)	
Other	0.87 (0.49–1.57)		1.26 (0.88–1.81)	
Age in years		0.31		<0.001
<20	0.62 (0.36–1.05)		0.35 (0.24–0.50)	
20–29	0.90 (0.72–1.13)		0.82 (0.71–0.94)	
30–39	1.00		1.00	
≥40	0.98 (0.57–1.69)		1.07 (0.75–1.52)	
Education		<0.001		0.04
No secondary education	0.43 (0.29–0.65)		0.66 (0.48–0.91)	
Secondary education	0.68 (0.53–0.86)		0.94 (0.80–1.11)	
Tertiary education	1.00		1.00	
Parity		0.04		<0.001
First child	1.26 (1.01–1.56)		0.53 (0.46–0.60)	
Subsequent child	1.00		1.00	
Pregnancy planning		0.004		0.02
Planned	1.38 (1.10–1.71)		1.19 (1.03–1.37)	
Unplanned	1.00		1.00	
Self-reported health status		<0.001		0.02
Poor/Fair	0.77 (0.53–1.11)		0.91 (0.70–1.18)	
Good	1.00		1.00	
Very good	1.57 (1.22–2.02)		1.13 (0.97–1.32)	
Excellent	1.41 (1.05–1.89)		1.29 (1.07–1.55)	
Household income ^b^		0.28		<0.001
>$150,000	0.99 (0.68–1.44)		0.90 (0.72–1.11)	
$100,001–150,000	1.02 (0.73–1.42)		1.09 (0.89–1.33)	
$70,001–100,000	1.00		1.00	
$50,001–70,000	0.89 (0.62–1.29)		1.58 (1.25–2.00)	
$30,001–50,000	0.75 (0.51–1.11)		1.26 (0.98–1.62)	
$20,001–30,000	0.59 (0.34–1.02)		0.83 (0.57–1.21)	
<$20,000	0.65 (0.34–1.24)		0.95 (0.61–1.48)	
Household deprivation ^c^		0.19		0.89
1–2 (least deprived)	1.51 (1.07–2.12)		0.98 (0.79–1.21)	
3–4	1.22 (0.89–1.68)		0.90 (0.73–1.10)	
5–6	1.28 (0.92–1.78)		0.97 (0.79–1.20)	
7–8	1.13 (0.83–1.54)		0.94 (0.77–1.16)	
9–10 (most deprived)	1.00		1.00	

Abbreviations: OR, odds ratio; CI, confidence interval. ^a^ Māori is New Zealand’s indigenous population, Other includes Middle Eastern, Latin American, and African. ^b^ Median household income in NZ in 2010 was $NZ 75,700 [27]. ^c^ Area-level socioeconomic deprivation was measured using the NZ Index of Deprivation [24].

**Table 4:** Independent associations of the demographics of partners with their intended infant feeding method and intended infant breastfeeding duration.

Partner Demographic Characteristics	OR (95% CI)	*p* Value	OR (95% CI)	*p* Value
**Self-prioritised ethnicity ^a^**		0.05		<0.001
European	1.00		1.00	
Māori	1.11 (0.80–1.53)		1.48 (1.17–1.86)	
Pacific Peoples	0.71 (0.55–0.93)		1.51 (1.22–1.88)	
Asian	1.19 (0.89–1.59)		1.69 (1.39–2.06)	
Other	1.04 (0.68–1.61)		1.61 (1.18–2.21)	
**Age in years**		0.07		<0.001
<20	0.51 (0.30–0.89)		0.28 (0.16–0.48)	
20–29	0.87 (0.71–1.08)		0.98 (0.84–1.14)	
30–39	1.00		1.00	
≥40	1.03 (0.78–1.35)		1.12 (0.92–1.35)	
**Education**		0.01		0.35
No secondary education	0.62 (0.45–0.85)		0.82 (0.63–1.08)	
Secondary education	0.89 (0.71–1.12)		1.00 (0.85–1.18)	
Tertiary education	1.00		1.00	
**Self-reported health status**		0.09		0.08
Poor/Fair	0.90 (0.69–1.19)		1.06 (0.86–1.31)	
Good	1.00		1.00	
Very good	1.15 (0.93–1.42)		1.09 (0.93–1.26)	
Excellent	1.37 (1.00–1.88)		1.32 (1.07–1.64)	
**Household income ^b^**		0.30		<0.001
> $150,000	0.95 (0.70–1.28)		0.61 (0.50–0.76)	
$100,001–150,000	0.83 (0.63–1.10)		0.82 (0.67–1.00)	
$70,001–100,000	1.00		1.00	
$50,001–70,000	1.15 (0.82–1.60)		1.35 (1.07–1.69)	
$30,001–50,000	0.88 (0.62–1.24)		1.44 (1.12–1.85)	
$20,001–30,000	0.92 (0.54–1.57)		1.43 (0.97–2.10)	
<$20,000	0.59 (0.33–1.05)		0.84 (0.52–1.36)	
**Household deprivation ^c^**		0.16		<0.001
1–2 (least deprived)	1.26 (0.95–1.67)		0.70 (0.57–0.86)	
3–4	1.24 (0.94–1.62)		0.73 (0.60–0.89)	
5–6	1.34 (1.01–1.78)		0.83 (0.67–1.02)	
7–8	1.36 (1.03–1.80)		1.04 (0.85–1.28)	
9–10 (most deprived)	1.00		1.00	

Abbreviations: OR, odds ratio; CI, confidence interval. ^a^ Māori is New Zealand’s indigenous population, Other includes Middle Eastern, Latin American, and African. ^b^ Median household income in NZ in 2010 was $NZ 75,700 [27]. ^c^ Area-level socioeconomic deprivation was measured using the NZ Index of Deprivation [24].

**Table 5:** Agreement between pregnant women and their partners on initial infant feeding method and breastfeeding duration for their infant.

Comparisons	Infant was Breastfed for >6 Months		Odds Ratio (95% CI) for Any Breastfeeding >6 Months		
	Yes *n =* 3793	No *n =* 2683	Univariate	Adjusted for Maternal Demographics ^a^	Adjusted for Partner Demographics ^b^
Parents whose infant feeding method intentions were concordant					
Parental agreement on only breastfeeding versus all other infant feeding intentions					
Both parents agreed on only breastfeeding, *n* (row %)	2169 (66)	1109 (34)	5.0 (3.3–7.7)	6.3 (3.9–10.2)	5.7 (3.6–9.2)
All other concordant infant feeding intentions, *n* (row %)	30 (28)	77 (72)	1.00	1.00	1.00
Parental agreement on breastfeeding duration					
Both parents agreed on breastfeeding for >6 months, *n* (row %)	1240 (77)	369 (23)	4.4 (3.5–5.4)	4.9 (3.9–6.2)	5.0 (4.0–6.3)
Both parents agreed on breastfeeding for ≤6 months, *n* (row %)	231 (44)	299 (56)	1.00	1.00	1.00
Parents whose infant feeding intentions were concordant or discordant					
Parental agreement on only breastfeeding versus all other infant feeding intentions					
Both parents agreed on only breastfeeding, *n* (row %)	2169 (66)	1109 (34)	2.6 (2.2–3.0)	2.8 (2.3–3.3)	2.6 (2.1–3.0)
All other infant feeding intentions both concordant and discordant, *n* (row %)	320 (43)	420 (57)	1.00	1.00	1.00
Parental agreement on breastfeeding duration					
Both parents agreed on breastfeeding for >6 months, *n* (row %)	1240 (77)	369 (23)	3.0 (2.6–3.5)	3.5 (2.9–4.1)	3.2 (2.8–3.8)
Partner has decided on breastfeeding for ≤6 months or hasn’t thought about duration, *n* (row %)	1027 (53)	921 (47)	1.00	1.00	1.00
Parental agreement on breastfeeding duration					
Both parents agreed on breastfeeding for >6 months, *n* (row %)	1240 (77)	369 (23)	4.7 (4.0–5.5)	5.0 (4.2–5.9)	4.7 (3.9–5.5)
Mother has decided on breastfeeding for ≤6 months, *n* (row %)	755 (42)	1056 (58)	1.00	1.00	1.00

Abbreviations: CI, confidence interval. ^a^ Self-prioritised ethnicity, age group in years, education, parity, pregnancy planning, self-reported health status, household income, and household socioeconomic deprivation. ^b^ Self-prioritised ethnicity, age group in years, education, self-reported health status, household income, and household socioeconomic deprivation.

## Supplementary Materials

The following are available online at http://www.mdpi.com/1660-4601/15/6/1230/s1.

## References

[1] World Health Organization (2003). Global Strategy for Infant and Young Child Feeding.

[2] Centres for Disease Control and Prevention, Breastfeeding Report Card United States 2014: CDC National Immunization Surveys 2012 and 2013, Data, 2011 Births. http://www.cdc.gov/breastfeeding/data/NIS_data/index.htm.

[3] Australian Institute of Health and Welfare (2011). 2010 Australian National Infant Feeding Survey: Indicator Results.

[4] McAndrew F., Thompson J., Fellows L., Large A., Speed M., Renfrew M.J. (2012). Infant Feeding Survey 2010.

[5] Castro T., Grant C., Wall C., Welch M., Marks E., Fleming C., Teixeira J., Bandara D., Berry S., Morton S. (2017). Breastfeeding indicators among a nationally representative multi-ethnic sample of New Zealand children. N. Z. Med. J..

[6] Forster D.A., McLachlan H.L., Lumley J. (2006). Factors associated with breastfeeding at six months postpartum in a group of Australian women. Int. Breastfeed. J..

[7] Amir L.H., Donath S.M. (2012). Maternal diet and breastfeeding: A case for rethinking physiological explanations for breastfeeding determinants. Early Hum. Dev..

[8] Meedya S., Fahy K., Kable A. (2010). Factors that positively influence breastfeeding duration to 6 months: A literature review. Women Birth.

[9] Giugliani E.R., Caiaffa W.T., Vogelhut J., Witter F.R., Perman J.A. (1994). Effect of breastfeeding support from different sources on mothers’ decisions to breastfeed. J. Hum. Lact..

[10] Bar-Yam N.B., Darby L. (1997). Fathers and breastfeeding: A review of the literature. J. Hum. Lact..

[11] Sharma M., Petosa R. (1997). Impact of expectant fathers in breast-feeding decisions. J. Am. Diet. Assoc..

[12] Schmidt M.M., Sigman-Grant M. (2000). Perspectives of low-income fathers’ support of breastfeeding: An exploratory study. J. Nutr. Educ..

[13] Scott J., Landers M., Hughes R., Binns C. (2001). Factors associated with breastfeeding at discharge and duration of breastfeeding. J. Paediatr. Child Health.

[14] Pisacane A., Continisio G.I., Aldinucci M., D’Amora S., Continisio P. (2005). A Controlled Trial of the Father’s Role in Breastfeeding Promotion. Pediatrics.

[15] Arora S., McJunkin C., Wehrer J., Kuhn P. (2000). Major Factors Influencing Breastfeeding Rates: Mother’s Perception of Father’s Attitude and Milk Supply. Pediatrics.

[16] Chezem J.C. (2012). Breastfeeding attitudes among couples planning exclusive breastfeeding or mixed feeding. Breastfeed. Med..

[17] Hounsome L., Dowling S. (2018). The mum has to live with the decision much more than the dad’; a qualitative study of men’s perceptions of their influence on breastfeeding decision-making. Int. Breastfeed. J..

[18] Mitchell-Box K., Braun K.L., Hurwitz E.L., Hayes D.K. (2013). Breastfeeding attitudes: Association between maternal and male partner attitudes and breastfeeding intent. Breastfeed. Med..

[19] Morton S.M.B., Atatoa Carr P.E., Grant C.C., Robinson E.M., Bandara D.K., Bird A., Ivory V.C., Kingi T.K.R., Liang R., Marks E.J. (2013). Cohort profile: Growing up in New Zealand. Int. J. Epidemiol..

[20] Morton S.M.B., Ramke J., Kinloch J.M., Grant C.C., Atatoa Carr P.E., Leeson H., Lee A.C., Robinson E. (2015). Growing Up in New Zealand cohort alignment with all New Zealand births. Aust. N. Z. J. Public Health.

[21] Morton S.M., Grant C.C., Carr P.E.A., Robinson E.M., Kinloch J.M., Fleming C.J., Kingi T.K.R., Perese L.M., Liang R. (2014). How do you recruit and retain a prebirth cohort? Lessons learnt from Growing Up in New Zealand. Eval. Health Prof..

[22] Morton S.M.B., Atatoa Carr P.E., Bandara D.K., Grant C.C., Ivory V.C., Kingi T.R., Liang R., Perese L.M., Peterson E., Pryor J.E. (2010). Growing Up in New Zealand: A Longitudinal Study of New Zealand Children and Their Families. Report 1: Before We Are Born.

[23] World Health Organization Indicators for Assessing Breast-Feeding Practices: Report of An Informal Meeting, 11–12 June 1991, Geneva, Switzerland. http://apps.who.int/iris/handle/10665/62134.

[24] Salmond C., Crampton P., Atkinson J. (2007). NZDep2006 Index of Deprivation.

[25] Bowling A. (2005). Just One Question: If One Question Works, Why Ask Several?. J. Epidemiol. Community Health.

[26] Landis J.R., Koch G.G. (1977). The measurement of observer agreement for categorical data. Biometrics.

[27] Perry B. (2011). Household Incomes in New Zealand: Trends in Indicators of Inequality and Hardship 1982 to 2010.

[28] World Health Organization, United Nations Children’s Fund (2014). Global Nutrition Targets 2025—Breastfeeding Policy Brief (WHO/NMH/NHD/14.7).

[29] World Health Organization Unicef, Baby-Friendly Hospital Initiative: Revised, Updated and Expanded for Integrated Care. www.who.int/nutrition/publications/infantfeeding/9789241594950/en/index.html.

[30] World Health Organization Global Data Bank on Infant and Young Child Feeding. http://www.who.int/nutrition/databases/infantfeeding/en/.

[31] Li R., Scanlon K.S., Serdula M.K. (2005). The validity and reliability of maternal recall of breastfeeding practice. Nutr. Rev..

[32] Grummer-Strawn L.M., Scanlon K.S., Fein S.B. (2008). Infant feeding and feeding transitions during the first year of life. Pediatrics.

[33] Hamade H., Chaaya M., Saliba M., Chaaban R., Osman H. (2013). Determinants of exclusive breastfeeding in an urban population of primiparas in Lebanon: A cross-sectional study. BMC Public Health.

[34] Lau Y. (2010). Breastfeeding intention among pregnant Hong Kong Chinese women. Matern. Child Health J..

[35] Boudet-Berquier J., Salanave B., Desenclos J.C., Castetbon K. (2017). Association between maternal prepregnancy obesity and breastfeeding duration: Data from a nationwide prospective birth cohort. Matern. Child Nutr..

[36] Kachoria R., Oza-Frank R. (2014). Differences in breastfeeding initiation by maternal diabetes status and race, Ohio 2006–2011. Matern. Child Health J..

[37] Lutsiv O., Pullenayegum E., Foster G., Vera C., Giglia L., Chapman B., Fusch C., McDonald S. (2013). Women’s intentions to breastfeed: A population-based cohort study. Int. J. Obstet. Gynaecol..

[38] Britton C., McCormick F.M., Renfrew M.J., Wade A., King S.E. (2007). Support for breastfeeding mothers. Cochrane Database Syst. Rev..

[39] Wolfberg A.J., Michels K.B., Shields W., O’campo P., Bronner Y., Bienstock J. (2004). Dads as breastfeeding advocates: Results from a randomized controlled trial of an educational intervention. Am. J. Obstet. Gynecol..

[40] Vaaler M.L., Castrucci B.C., Parks S.E., Clark J., Stagg J., Erickson T. (2011). Men’s attitudes toward breastfeeding: Findings from the 2007 Texas Behavioral Risk Factor Surveillance System. Matern. Child Health J..

[41] Özlüses E., Çelebioglu A. (2014). Educating fathers to improve breastfeeding rates and paternal-infant attachment. Indian Pediatrics.

[42] Sipsma H.L., Jones K.L., Cole-Lewis H. (2015). Breastfeeding among adolescent mothers: A systematic review of interventions from high-income countries. J. Hum. Lact..

[43] Acharya P., Khanal V. (2015). The effect of mother’s educational status on early initiation of breastfeeding: Further analysis of three consecutive Nepal Demographic and Health Surveys. BMC Public Health.

[44] Avery A.B., Magnus J.H. (2011). Expectant fathers’ and mothers’ perceptions of breastfeeding and formula feeding: A focus group study in three US cities. J. Hum. Lact..

[45] Henderson L., McMillan B., Green J.M., Renfrew M.J. (2011). Men and infant feeding: Perceptions of embarrassment, sexuality, and social conduct in white low-income British men. Birth.

[46] Morton Consortium (2006). Report 2: Proposed Longitudinal Study Design.

[47] Morton Consortium (2006). Report 1: Conceptual Framework and Vision.

