# Supplementary material for: Agreement between Future Parents on Infant Feeding Intentions and Its Association with Breastfeeding Duration: Results from the Growing Up in New Zealand Cohort Study
Source: Int J Environ Res Public Health. 2018 Jun 11;15(6):1230. doi: 10.3390/ijerph15061230 (PMC6025038; doi:10.3390/ijerph15061230)
Supplement: Supplementary file 1 [file ijerph-15-01230-s001.pdf]

International Journal of *Environmental Research and Public Health*

MDPI

Supplementary Material:

Table S1. Demographic characteristics of enrolled pregnant women and their partners.

Variable	Pregnant Women n = 4397 n (Col %)	Partner n = 4397 n (Col %)
Self-prioritised ethnicity [a]		
European	2687 (61)	2655 (61)
Māori	439 (10)	433 (10)
Pacific Peoples	426 (10)	496 (11)
Asian	670 (15)	587 (13)
Other	166 (4)	214 (5)
Age in years		
<20	133 (3)	84 (2)
20–29	1563 (36)	1159 (26)
30–39	2511 (57)	2509 (57)
≥40	190 (4)	644 (15)
Education		
No secondary education	192 (4)	300 (7)
Secondary education	925 (21)	897 (20)
Tertiary education	3274 (75)	3195 (73)
Parity		
First child	1972 (45)	-
Subsequent child	2425 (55)	-
Pregnancy planning		
Planned	3023 (69)	-
Unplanned	1363 (31)	-
Self-reported health status		
Poor/Fair	327 (7)	535 (13)
Good	1378 (31)	1675 (40)
Very good	1715 (39)	1428 (34)
Excellent	973 (22)	515 (12)
Household income [b]		
> $150,000	616 (17)	726 (19)
$100,001–150,000	927 (25)	902 (24)
$70,001–100,000	890 (24)	873 (23)
$50,001–70,000	581 (16)	599 (16)
$30,001–50,000	443 (12)	451 (12)
$20,001–30,000	136 (4)	143 (4)
<$20,000	99 (3)	87 (2)
Household socioeconomic deprivation [c]		
1–2 (least deprived)	825 (19)	825 (19)
3–4	891 (20)	894 (20)
5–6	820 (19)	813 (19)
7-8	911 (21)	910 (21)
9–10 (most deprived)	948 (22)	953 (22)

[a] Māori is New Zealand's indigenous population, Other includes Middle Eastern, Latin American and African. [b] Median household income in NZ in 2010 was $NZ 75,700 [27]. [c] Area-level socioeconomic deprivation was measured using the NZ Index of Deprivation [24]. **-** Not asked of partners.

International Journal of
Environmental Research
and Public Health

Table S2. Maternal and partner demographics by intended infant feeding method (Breast vs. Other Methods) and intended breastfeeding duration (> 6 Months vs. ≤ 6 Months).

	Intended Infant Feeding Method						Intended Breastfeeding Duration					
	Mothers			Partners			Mothers			Partners		
**Demographic characteristic**	Breast feeding only	Other*	*P* Value	Breast	Other	*P* Value	≤6 Months n (Row %)	>6 Months n (Row %)	*P* Value	≤6 Months n (Row %)	>6 Months n (Row %)	*P* Value
**Self-prioritised ethnicity** [a]			<0.001			0.05			<0.001			<0.001
European	3054 (92)	271 (8)		2184 (87)	318 (13)		1093 (34)	2160 (66)		1051 (48)	1144 (52)	
Māori	733 (86)	119 (14)		367 (88)	48 (12)		239 (30)	569 (70)		137 (38)	221 (62)	
Pacific Peoples	709 (81)	169 (19)		388 (83)	79 (17)		222 (27)	602 (73)		155 (38)	256 (62)	
Asian	777 (87)	115 (13)		490 (89)	60 (11)		298 (34)	574 (66)		182 (35)	333 (65)	
Other	199 (89)	25 (11)		179 (88)	25 (12)		53 (24)	166 (76)		65 (36)	114 (64)	
**Age in years**			0.07			0.06			<0.001			<0.001
<20	259 (86)	44 (15)		62 (79)	17 (22)		155 (54)	133 (46)		47 (73)	17 (27)	
20 – 29	2129 (88)	293 (12)		959 (86)	154 (14)		781 (33)	1558 (67)		420 (44)	541 (56)	
30 – 39	2879 (90)	336 (11)		2067 (88)	290 (12)		908 (29)	2218 (71)		906 (43)	1191 (57)	
40+	214 (89)	27 (11)		527 (88)	72 (12)		66 (28)	167 (72)		220 (41)	323 (60)	
**Education**			<0.001			0.01			0.35			0.35
Primary	330 (78)	94 (22)		232 (82)	52 (18)		133 (35)	251 (65)		111 (48)	120 (52)	
Secondary	1267 (86)	205 (14)		732 (87)	114 (14)		463 (33)	959 (67)		324 (43)	426 (57)	
Tertiary	3873 (91)	400 (9)		2648 (88)	367 (12)		1308 (31)	2860 (69)		1158 (43)	1524 (57)	
**Parity**			<0.001			-			<0.001			-
First child	2356 (91)	226 (9)		-	-		1011 (40)	1507 (60)		-	-	
Subsequent child	3125 (87)	474 (13)		-	-		899 (26)	2569 (74)		-	-	
**Pregnancy planning**			<0.001			-			0.03			-
Planned pregnancy	3374 (91)	346 (9)		-	-		1121 (31)	2517 (69)		-	-	
Unplanned pregnancy	2091 (86)	350 (14)		-	-		780 (34)	1550 (67)		-	-	
**Self-reported health status**			<0.001			0.09			0.004			0.09
Poor/ Fair	536 (84)	100 (16)		454 (85)	79 (15)		210 (35)	384 (65)		197 (44)	254 (56)	
Good	1794 (86)	301 (14)		1447 (86)	228 (14)		680 (34)	1336 (66)		666 (45)	807 (55)	
Very good	2024 (92)	180 (8)		1252 (88)	172 (12)		674 (31)	1493 (69)		549 (43)	723 (57)	
Excellent	1123 (91)	118 (10)		462 (90)	53 (10)		344 (29)	862 (72)		180 (39)	287 (62)	
**Household Income** [b]			0.001			.32			0.005			<0.001
> $150,000	672 (90)	73 (10)		635 (88)	89 (12)		261 (36)	468 (64)		352 (54)	296 (46)	
$100,001-150,000	1057 (91)	99 (9)		778 (86)	124 (14)		361 (32)	778 (68)		368 (47)	412 (53)	
$70,001-100,000	1087 (91)	111(9)		770 (88)	102 (12)		393 (34)	782 (67)		328 (42)	449 (58)	
$50,001-70,000	764 (89)	96 (11)		536 (90)	62 (10)		223 (27)	607 (73)		186 (35)	343 (65)	

$30,001-50,000	640 (87)	98 (13)		392 (87)	59 (13)		208 (29)	500 (71)		137 (34)	271 (66)	
$20,001-30,000	247 (85)	45 (15)		125 (87)	18 (13)		94 (33)	188 (67)		45 (34)	88 (66)	
<$20,000	185 (84)	35 (16)		72 (82)	16 (18)		64 (31)	145 (69)		34 (46)	40 (54)	
**Household socioeconomic deprivation** [c]			<0.001			0.16			0.34			<0.001
1-2 (least deprived)	913 (92)	77 (8)		671 (88)	96 (13)		329 (34)	640 (66)		335 (48)	357 (52)	
3-4	1023 (90)	114 (10)		740 (87)	108 (13)		363 (33)	744 (67)		352 (47)	393 (53)	
5-6	965 (90)	105 (10)		678 (88)	91 (12)		335 (32)	720 (68)		303 (44)	382 (56)	
7-8	1136 (89)	143 (11)		756 (88)	100 (12)		395 (32)	841 (68)		285 (39)	454 (61)	
9-10 (most deprived)	1442 (85)	261 (15)		766 (85)	138 (15)		488 (30)	1129 (70)		316 (40)	483 (61)	

[*] Other includes: All other infant feeding methods including mixed feeding. [a] Māori is New Zealand's indigenous population, Other includes Middle Eastern, Latin American and African. [b] Median household income in NZ in 2010 was $NZ 75,700 [27] [c] Area-level socioeconomic deprivation was measured using the NZ Index of Deprivation [24].
